# Measurement Sensitivity Improvement of All-Optical Atomic Spin Magnetometer by Suppressing Noises

**DOI:** 10.3390/s16060896

**Published:** 2016-06-17

**Authors:** Xiyuan Chen, Hong Zhang, Sheng Zou

**Affiliations:** 1 School of Instrument Science and Engineering, Southeast University, Nanjing 210096, China; 230129459@seu.edu.cn (H.Z.); 230129461@seu.edu.cn (S.Z.); 2 Key Laboratory of Micro-Inertial Instrument and Advanced Navigation Technology Ministry of Education, Nanjing 210096, China

**Keywords:** all optical, atomic spin magnetometer, noise, sensitivity

## Abstract

Quantum manipulation technology and photoelectric detection technology have jointly facilitated the rapid development of ultra-sensitive atomic spin magnetometers. To improve the output signal and sensitivity of the spin-exchange-relaxation-free (SERF) atomic spin magnetometer, the noises influencing on the output signal and the sensitivity were analyzed, and the corresponding noise suppression methods were presented. The magnetic field noises, including the residual magnetic field noise and the light shift noise, were reduced to approximately zero by employing the magnetic field compensation method and by adjusting the frequency of the pump beam, respectively. With respect to the operation temperature, the simulation results showed that the temperature of the potassium atomic spin magnetometer realizing the spin-exchange relaxation-free regime was 180 °C. Moreover, the fluctuation noises of the frequency and the power were suppressed by using the frequency and the power stable systems. The experimental power stability results showed that the light intensity stability was enhanced 10%. Contrast experiments on the sensitivity were carried out to demonstrate the validity of the suppression methods. Finally, a sensitivity of 13 fT/Hz^1/2^ was successfully achieved by suppressing noises and optimizing parameters.

## 1. Introduction

In recent years, sensitive magnetometers have been used in many fields, such as biomedicine [1,2], Magnetic Resonance Imaging (MRI) [3,4], material science [5,6], geography [7,8], basic physics [9,10,11,12] and magnetic induction tomography [13]. With the rapid developments of quantum manipulation technology and photoelectric detection technology, the spin-exchange relaxation-free (SERF) atomic magnetometer [14] with ultrahigh theoretical sensitivity has received considerable attention, and it has been a hot research area [15,16,17,18].

Romalis group of Princeton University was the first to realize a SERF atomic spin magnetometer [19]. This group successfully achieved a sensitivity of 0.16 fT/Hz^1/2^ in 2010, which currently still is the highest sensitivity achieved in magnetic field measurement [20]. Kamada *et al.* have used atomic magnetometers with optical gradiometer configurations to improve the sensitivity [21]. Atomic spin magnetometers are expected to be a new developmental direction in the future, however, although the SERF atomic spin magnetometer has been successfully implemented, research on noise suppression methods based on constructing an output signal model and analyzing the noise sources and noises are still vitally important.

A SERF atomic spin magnetometer mainly consists of an optical pumping system, optical detection system, alkali-metal cell, magnetic shielding and magnetic compensation system, and non-magnetic electric heating system. All of these parts can produce noises. In this paper, according to the Bloch equation, the output signal was established. The factors influencing the sensitivity, such as magnetic field, temperature and light field were analyzed; meanwhile, corresponding suppression methods and the optimal operation temperature were proposed. Finally, the magnetic field measurement sensitivities were analyzed by contrast experiments.

## 2. Principle of Atomic Spin Magnetometer

**Figure 1:**
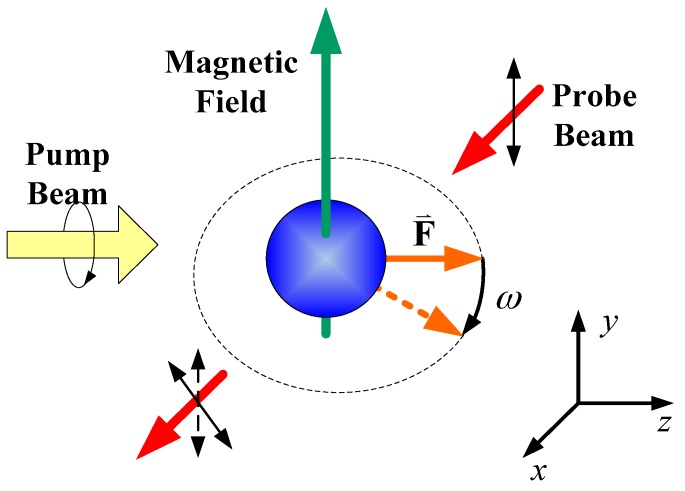
Principle of the SERF atomic spin magnetometer.

The principle of the SERF atomic spin magnetometer is shown in Figure 1. Optical pumping (OP) [22,23] technology is used to polarize electron spin, and the spin angular momentum can be transferred to the nuclei via collisions. A circularly-polarized light, whose wavelength is tuned to the D1 resonance of the alkali metal atoms, can be used to polarize the atomic spin. A magnetic field perpendicular to the pump beam can induce a Larmor procession. At this time, a linearly-polarized light perpendicular to the plane of the magnetic field and pump beam can detect optical rotation which reflects such a Larmor procession. Realizing SERF regime thus needs three essential conditions, including a weak magnetic field, high heating temperature and optical pumping.

**Figure 2:**
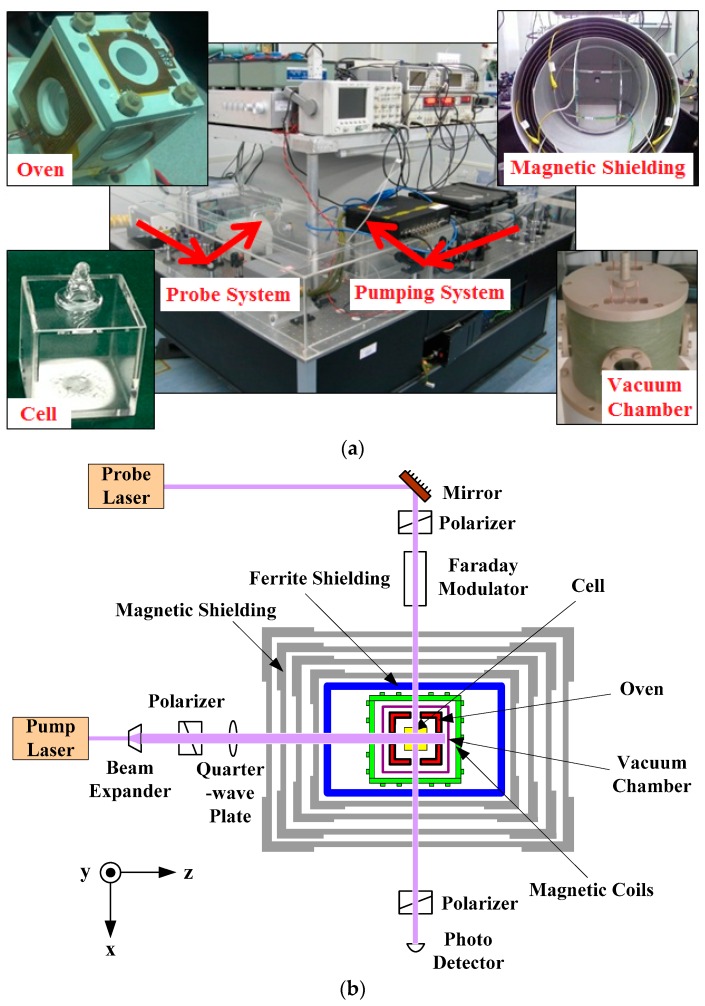
Arrangement of the SERF atomic spin magnetometer. (**a**) The photograph of the experimental device; (**b**) The schematic diagram of the light path. The pump beam propagates along the *z* direction. The probe beam is perpendicular to the pump beam and propagates along the *x* direction. The ferrite shielding is used to reduce the magnetic noise.

The arrangement of the SERF atomic spin magnetometer is shown in Figure 2. A cubic glass cell (25 mm length) contains a droplet of K atoms, 2 atm of ^4^He to reduce the wall collision relaxation, and 30 Torr of N_2_ to quench the alkali-metal excited state. The cell is heated to more than one hundred of degrees by a non-magnetic electric heating device to obtain a high atomic number density. The circularly-polarized beam is tuned to approximately 770.108 nm. The center wavelength of the linearly-polarized probe beam is about 766.2 nm. A Faraday modulator is placed in the probe optical path. Optical rotation can be detected by this linearly-polarized probe beam.

A four-layer magnetic shielding with high permeability is employed to reduce the ambient magnetic field. A tri-axial magnetic coils can produce a uniform magnetic field along the *x*, *y* and *z* directions, respectively. A vacuum chamber is used in our experiments to isolate the heat conduction from the oven due to the limitation on performance of the magnetic shielding caused by the temperature.

## 3. Output Signal of SERF Atomic Spin Magnetometer

### 3.1. Output Signal of SERF Atomic Spin Magnetometer

The evolution of the electron spin polarization can be well expressed by the Bloch equation [24]:

(1) $$\frac{\partial {\vec{P}}^{e}}{\partial t}=\frac{1}{q}(\gamma^{e}\vec{B}\times {\vec{P}}^{e}+R_{p}(\hat{z}-{\vec{P}}^{e})+R_{m}(\hat{x}-{\vec{P}}^{e})-R_{\text{sd}}{\vec{P}}^{e})$$

(2) $$\frac{\partial {\vec{P}}^{e}}{\partial t}=\frac{1}{q}(\gamma^{e}\vec{B}\times {\vec{P}}^{e}+R_{p}(\hat{z}-{\vec{P}}^{e})+R_{m}(\hat{x}-{\vec{P}}^{e})-R_{\text{sd}}{\vec{P}}^{e})$$

where ${\vec{P}}^{e}$is the electron spin polarization, *q* is the slowing-down factor, γ^e^ is the electron gyromagnetic ratio, $\vec{B}$ is the external magnetic field in the magnetic shielding, *R*_p_ is the pumping rate of the pump beam defined as the average rate at which an unpolarized atom absorbs a photon from the pump beam, *R*_m_ is the pumping rate of the probe beam caused by the small circular component in the probe beam, *R*_sd_ is the spin-destruction relaxation rate, and $\hat{z}$ and $\hat{x}$ are the unit vectors of the *z* direction and the *x* direction, respectively. $R_{\mathrm{sd}}^{a–a}$ is the spin-destruction relaxation rate between the alkali metal atoms, $R_{\mathrm{sd}}^{a–g}$ is the spin-destruction relaxation rate between the alkali metal atoms and the gases including the noble and quenching gases, and *R*_D_ is the spin diffusion relaxation caused by the collision with the wall of the cell. Under the conditions of the magnetic field existing and changing slowly, by setting the left side of Equation (1) to zero, the steady-state solution in the *x* direction can be considered as [24]:

(3) $$P_{x}^{e}=\frac{R_{P}(\beta_{y}+\beta_{x}\beta_{z})+R_{m}(1+\beta_{x}^{2})}{R_{\text{tot}}(1+\beta_{x}^{2}+\beta_{y}^{2}+\beta_{z}^{2})}$$

where $\vec{\beta }=\gamma^{e}\vec{B}/R_{\text{tot}}$ is the magnetic field parameter, $\left|\vec{\beta }\right|\ll 1$, and *R*_tot_ is the total relaxation rate. The output signal of the SERF atomic spin magnetometer can be obtained by applying the lock-in amplifier to extract the first harmonic, and the signal is proportional to the optical rotation angle θ [25]:

(4) $$I=I_{0}\;\sin^{2}[\theta +\alpha \sin(\omega_{\mathrm{mod}}t)]$$

(5) $$S_{\text{out}}=2I_{0}\theta \alpha$$

(6) θ=π2lnrecfPxe{−Im[V(ν−νD1)]+Im[V(ν−νD2)]}

where *I*_0_ is the light intensity, which is detected by the photoelectric detector placed in the *x* direction, α is the modulation amplitude of the Faraday modulator, ω_mod_ is the modulation frequency, *l* is the distance of the light propagating through the alkali-metal cell, *n* is the density number of the alkali-metal atoms, which is closely related to the temperature *T* in the cell, *r*_e_ is the electron classical radius, *c* is the speed of light, *f* is the oscillator strength, *V* is the Voigt profile, and *ν*_D1_ and *ν*_D2_ are the resonance frequencies of the D1 and the D2 transitions, respectively. Equations (3) and (6) are inserted into Equation (5), it can be obtained:

(7) $$S_{\text{out}}=\alpha I_{0}nK\frac{R_{P}(\beta_{y}+\beta_{x}\beta_{z})+R_{m}(1+\beta_{x}^{2})}{R_{\text{tot}}(1+\beta_{x}^{2}+\beta_{y}^{2}+\beta_{z}^{2})}$$

(8) K=πlrecf{−Im[V(ν−νD1)]+Im[V(ν−νD2)]}

where *K* is defined as the calibration coefficient. Equation (7) is described as the output signal.

### 3.2. Noises Influencing on Performance of SERF Atomic Spin Magnetometer

The above output signal indicates that the magnetic field included in the magnetic field parameter $\vec{\beta }=\gamma^{e}\vec{B}/R_{\text{tot}}$, the temperature which directly impacts the density number of the alkali metal atoms and the relaxation rate, and the light which can affect the pumping rate and the light shift, are the main noise sources influencing on the output signal and the sensitivity of the SERF atomic spin magnetometer. Thus, analyzing and suppressing the noises and optimizing the operation temperature are very necessary.

#### 3.2.1. Magnetic Field Noise

**Figure 3:**
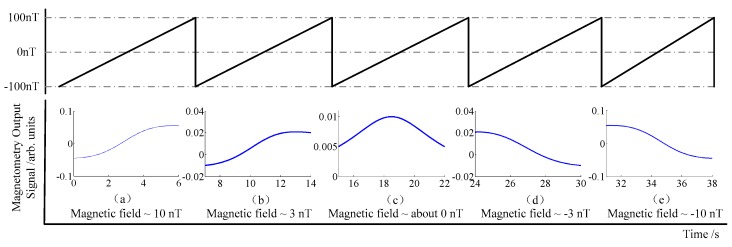
Simulation of the magnetic field compensation process. The magnetic field is changing from −100 nT to 100 nT by scanning the sawtooth wave.

Light shift referring to the AC Stark shift is equivalent to a virtual magnetic field. The magnetic field including the residual magnetic field in the magnetic shielding and the light shift is one of the noise sources restricting the sensitivity. The SERF magnetometer works at a weak magnetic environment, so a larger residual magnetic field may cause a low sensitivity, and even make the magnetometer not work normally. Fortunately, the residual magnetic field can be compensated by the magnetic field produced by the magnetic coils. According to the steady state Equation (3), the simulation of the magnetic field compensation process is shown in Figure 3.

As Figure 3 shown, the method and the steps of the residual magnetic field compensation are described as follows:

1. During the simulation process, the residual magnetic field in the *x* direction is assumed as 10 nT, as shown in Figure 3a. A changing magnetic field from −100 nT to 100 nT is scanning in the *z* direction, meanwhile, the offset of the magnetic field in the *x* direction needs to be adjusted synchronously. By observing the output signal, it can be known that if the residual magnetic field in the shielding is counteracted gradually, the output signal will reduce, as shown in Figure 3b; otherwise, the output signal will increase. Continue to adjust the offset of the magnetic field in the *x* direction until the output signal reaches the minimum value, as shown in Figure 3c. After passing through this minimum value, the pattern of the output signal overturns, presently, the signal’s amplitude increases gradually, as shown in Figure 3d,e, respectively. The minimum value of the output signal is considered as the compensation point in the *x* direction.
2. In a similar way, the scanning field condition of the *z* direction is kept invariant and the offset of the magnetic field in the *y* direction adjusted to achieve the minimum output value in this direction.
3. After compensating the magnetic field in the *x* and *y* directions by using steps 1 and 2, a scanning field sawtooth wave from −100 nT to 100 nT is applied to the *x* direction. The offsets of the magnetic field in the *z* and *y* directions are adjusted until the minimum output values are achieved, respectively.
4. Steps 1 to 3 are repeated until the offset values of three directions are approximately constant. By using this compensation method, the residual magnetic field in the three directions can be counteracted, and the residual magnetic field noise can also be suppressed.

The energy levels can be shifted by the light field via the AC Stark shift, and this is known as the light shift. The atomic energy level shift, which originates from the interaction between the alkali metal spins and the applied AC electric field from the circularly-polarized light, is equivalent to a Zeeman energy shift due to a fictitious magnetic field in the direction of the photon spin, and it is impacted by the power and the frequency of the pump laser [24,25],

(9) L=−πrecfΦγeIm[V(ν−ν0)]s

where *L* is the virtual magnetic field, $\Phi =\frac{P}{h\nu }$ is the luminous flux, which is closely related with the intensity *P* and the frequency *ν* of the pump laser, *h* is the Planck constant, *ν*_0_ is the frequency at the resonant peak of the alkali-metal atom, and *s* is the photon spin.

**Figure 4:**
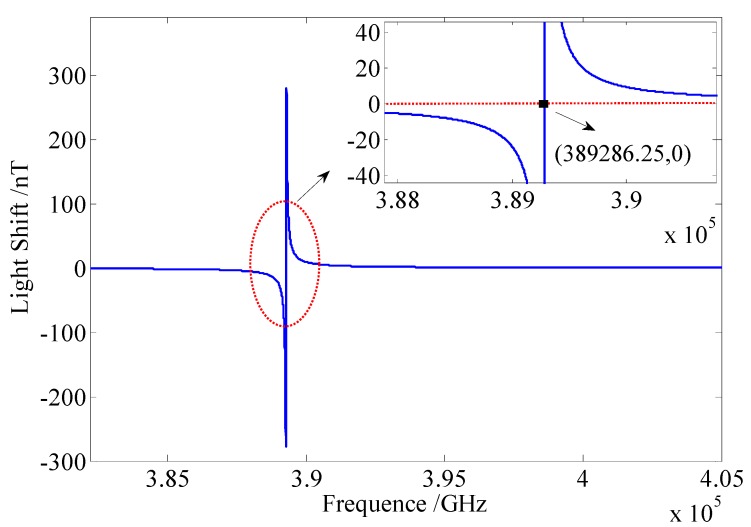
Simulation of the relation between the light shift and the frequency of the pump laser. The power of the pump laser is 100 mW, and the frequency at the resonant peak of potassium atom is 389,286.25 GHz.

Figure 4 shows that the simulation of the relation between the light shift and the frequency of the pump beam. With changing frequency, the light shift can pass through a zero point, which is at the resonant peak of 389,286.25 GHz.

#### 3.2.2. Temperature Optimization

The alkali-metal atomic density number and the total relaxation depend directly on the temperature in the cell. On the one hand, with increasing temperature, the atomic number density increases rapidly; on the other hand, the spin-destruction relaxation rate and the diffusion relaxation rate to walls can be directly increased due to the faster thermal velocity at a high temperature. Thus, the total relaxation rate increases. The greater the total relaxation rate is, the lower the atomic spin magnetometer’s sensitivity will be. Therefore, optimizing the heating temperature is particularly important.

**Figure 5:**
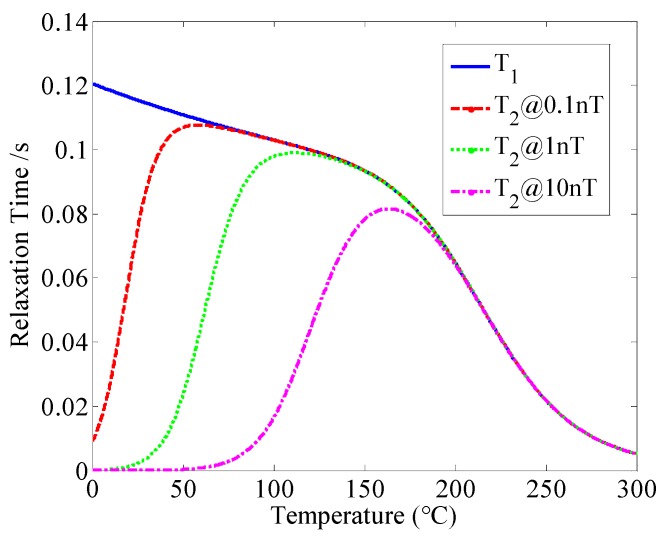
Simulation of the SERF atomic spin magnetometer realizing the SERF regime.

Take the potassium atomic magnetometers as examples. Generally, the electron spin relaxation time includes the transverse relaxation time *T*_2_ and the longitudinal relaxation time *T*_1_. The slowing-down factor *q* affects the relaxation. The relationships among these relaxation times are shown as follows [19]:

(10) $$\frac{1}{T_{1}}=\frac{1}{q}\left(R_{\text{sd}}+R_{P}+R_{m}\right)$$

(11) $$\frac{1}{T_{2}}=\frac{1}{T_{1}}+\frac{1}{q_{\text{se}}}R_{\text{se}}+R_{\text{gr}}$$

(12) $$\frac{1}{q_{\text{se}}}=\frac{2I(2I-1)}{3{\left(2I+1\right)}^{2}}$$

(13) $$R_{\text{tot}}=\frac{1}{T}10^{21.866+A_{K}-B_{K}/T}\cdot \sigma_{K-K}\cdot \sqrt{\frac{8k_{B}T}{\pi M_{K-K}}}+\frac{P_{\text{He}}}{k_{B}T}\cdot \sigma_{K-\mathrm{He}}\cdot \sqrt{\frac{8k_{B}T}{\pi M_{K-\mathrm{He}}}}+\frac{P_{N_{2}}}{k_{B}T}\cdot \sigma_{K-N_{2}}\cdot \sqrt{\frac{8k_{B}T}{\pi M_{K-N_{2}}}}+R_{P}+R_{m}$$

(14) $$R_{\text{se}}=\frac{{\left(\gamma^{e}B\right)}^{2}[q^{2}-{\left(2I+1\right)}^{2}]}{2n\nu_{e}\sigma_{\text{se}}^{e}q^{2}}$$

where $\frac{1}{q_{\text{se}}}$ is the spin-exchange broadening factor, $R_{\text{se}}$ is the spin-exchange relaxation rate, $R_{\text{gr}}$ is the relaxation due to the magnetic field gradient across the vapor cell, which is a tiny amount that is neglected due to the cell placement in the center of a uniform magnetic field, *I* is the nuclear spin quantum number, *T* is the temperature in cell, *A*_k_ = 4.402 and *B*_k_ = 4453 are the parameters, σ_species_ is the effective collisional cross-section, *K*_B_ is the Boltzmann constant, *M* is the reduced mass, *P*_species_ is the gas pressure, *v*_e_ is the relative thermal velocity between the alkali metals atoms, and $\sigma_{\text{se}}^{e}$ is the spin exchange collision cross section. One of the most striking features of the electron spin in the SERF regime is that the spin exchange is suppressed. In other words, if the spin-exchange relaxation rate *R*_se_ is suppressed, the transverse relaxation time *T*_2_ will be approximately equal to the longitudinal relaxation time *T*_1_, and the atomic spin SERF regime will be realized. According to Equations (10)–(14), we know that both the transverse relaxation time *T*_1_ and the longitudinal relaxation time *T*_2_ are related to the temperature *T*. The simulation of the atomic magnetometer realizing the SERF regime is shown in Figure 5.

Under the different magnetic field environment, with increasing temperature, the transverse relaxation time *T*_2_ and the longitudinal relaxation time *T*_1_ both overlap, which means that the magnetometer realizes the SERF regime. Figure 5 indicates that under the magnetic field of 1 nT, the temperature realizing the SERF regime is approximately 180 °C.

#### 3.2.3. Light Field Noise

**Figure 6:**
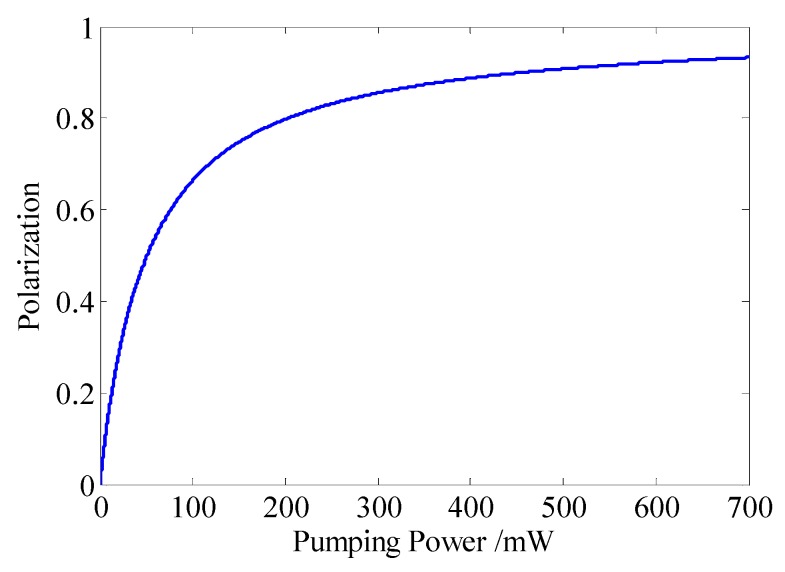
Relationship between the pumping power and the polarization.

In this paper, we mainly consider the fluctuations of the frequency and the power from the pump beam, which can cause the pumping rate, the polarization rate projection and the relaxation rate to be output by the forms of noises. In addition, the laser frequency fluctuation also can cause the light shift, as shown in Figure 6, where when the pumping power fluctuates from 20 mW to 25 mW, the polarization transforms from 28% to 33%.

**Figure 7:**
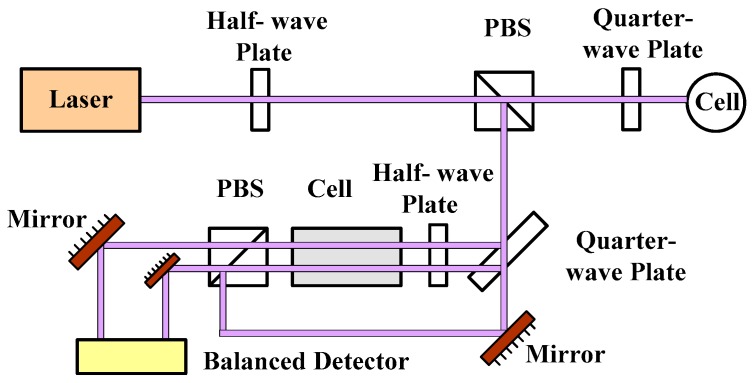
Schematic diagram of the frequency stabilization based on the saturation absorption method.

**Figure 8:**
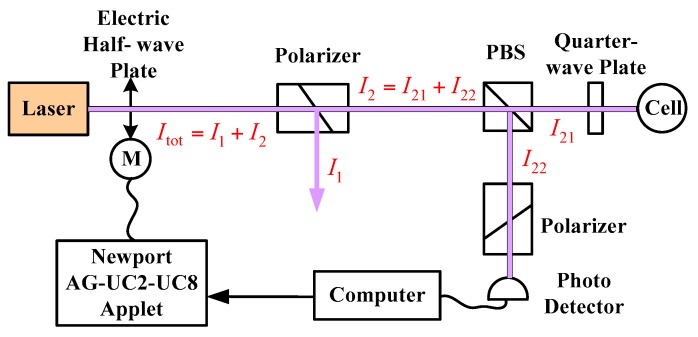
Schematic diagram of the power stabilization system. Newport AG-UC2-UC8 Applet is the piezo motor driven components.

**Figure 9:**
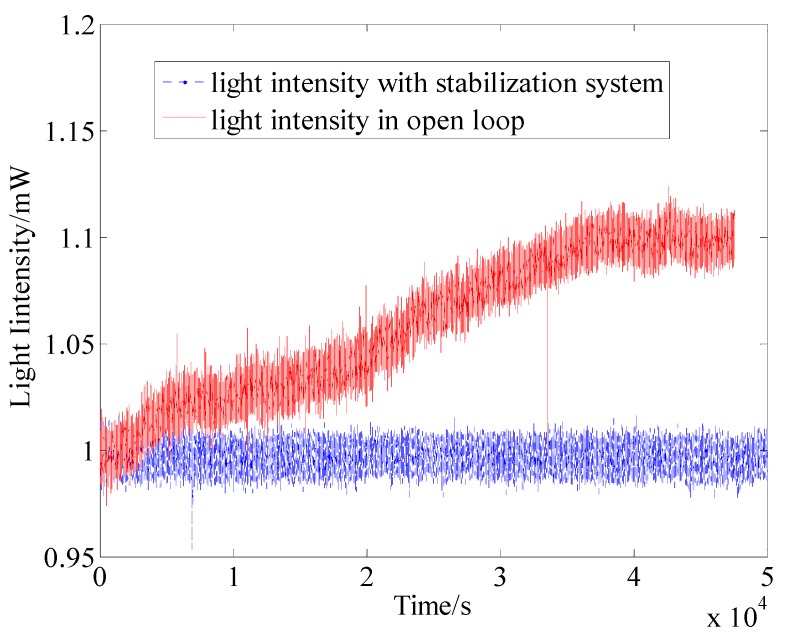
Experiment results of the light intensity stability.

If the pumping power fluctuates from 95 mW to 100 mW, the polarization will change from 65% to 66%. It is very important to stabilize the pumping power, especially in the low pumping power condition. According to Equation (9) and Figure 4, we can know that if the frequency fluctuation transforms to 472 GHz, the light shift changes about 4 nT. However, these two noises can be suppressed by employing the frequency and the power stabilization systems, as shown in Figure 7 and Figure 8, respectively.

(15) $$I_{\text{tot}}=I_{1}+I_{2},\;I_{2}=I_{21}+I_{22}$$

where *I*_tot_ is the total light intensity, which is the sum of light intensities *I*_1_ and *I*_2_. *I*_1_ is the light intensity with fluctuation, and *I*_2_ is the stable light intensity. The stable light intensity is divided into two beams *I*_21_ and *I*_22_, which are used to pump the atomic spin and monitor and stabilize the light intensity, respectively. The light intensity is transmitted to the computer by the photoelectric detector. The stable *I*_2_ can be stabilized through controlling the electric wave plate by the PID algorithm, while, the fluctuation noise is allotted to *I*_1_. Hence, the stable light intensity is successfully achieved. The experimental result of the light intensity stability is shown in Figure 9.

Figure 9 shows that the power stabilization system could effectively suppress the power fluctuation. Compared with these two test results, the light intensity had a 10% fluctuation without the power stabilization system.

## 4. Experimental and Analysis

**Figure 10:**
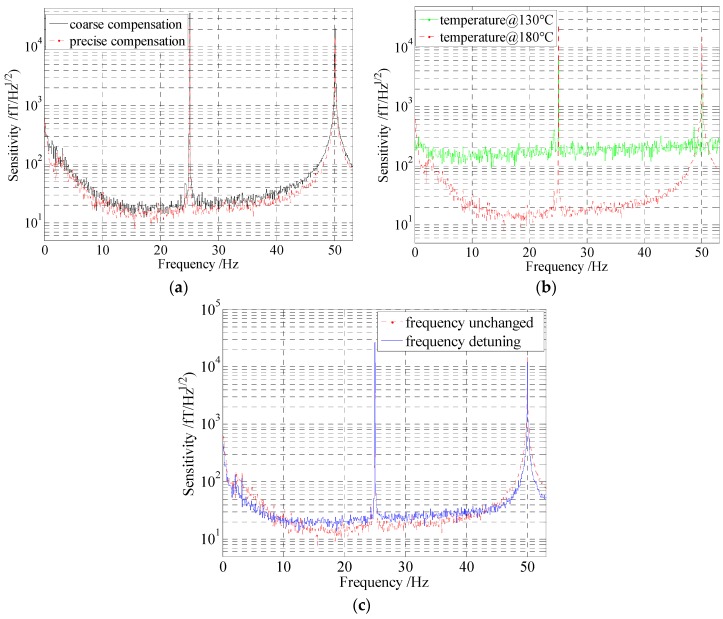
Experimental results of sensitivity comparison under the different situations. For each test, only one condition was changed. The dotted line acted as a comparison benchmark. (**a**) Optimization results of the residual magnetic field compensation in the magnetic shielding; (**b**) Optimization results of the temperature in cell; (**c**) Comparison results of the frequency detuning. On the basis of the un-optimized frequency, the frequency detuning was approximately 40 GHz.

The experimental apparatus is described as Figure 2. In order to analyze the effects of the noises on the sensitivity, relevant experiments were implemented. A 22.5 pT calibration signal at 25 Hz was applied to the *y* direction in these experiments. After FFT, the output signals were processed by divided a normalized frequency-response function with a fit to *A*/($f_{0}^{2}$ + $B_{\mathrm{bandwidth}}^{2}$)^1/2^, where *A* is the coefficient, *f*_0_ is the frequency, and *B*_bandwidth_ is the bandwidth [17]. By analyzing the signal to noise ratio, the sensitivities of the magnetometer under the different situations were achieved. The experimental results are shown in Figure 10. Figure 10a indicates that under the condition of the residual magnetic field coarse compensation, the sensitivity was about 20 fT/Hz^1/2^. However, when the residual magnetic field is compensated accurately, the sensitivity can be promoted efficiently, as shown by the dotted line in Figure 10a.

Figure 10b shows that at 130 °C, the sensitivity was about 150 fT/Hz^1/2^, it was much lower than that at the temperature in the SERF regime (180 °C).

**Figure 11:**
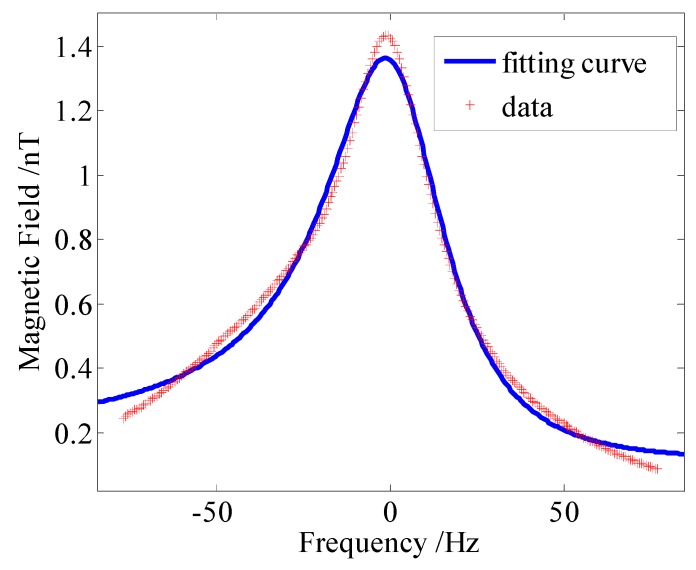
Test result of the light shift *L*_z_. The pumping power was 50 mW.

Figure 10c illustrates that at the unoptimized frequency, which is detuned approximately 40 GHz, the sensitivity was about 22 fT/Hz^1/2^. This is mainly attributed to the light shift, which was caused by the frequency detuning. Equation (3) is simplified and rewritten as:

(16) $$P_{x}^{e}=\frac{R_{P}\left[\left(B_{z}-L_{z}\right)B_{x}+\varepsilon B_{y}\right]}{R_{\text{tot}}\left[{\left(B_{z}-L_{z}\right)}^{2}+\varepsilon^{2}+B_{x}^{2}+B_{y}^{2}\right]}$$

where ε = *R*_tot_/γ^e^, *B*_x_, *B*_y_ and *B*_z_ are the total magnetic fields in the *x*, *y* and *z* directions, respectively, *B*_resz_ is the residual magnetic field, *B*_resz_ = *B*_z_ − *L*_z_, and *L*_z_ is the light shift in the *z* direction. According to the following fitting formula, the light shift can be obtained:

(17) $$y=a\frac{(x-b)e+cB_{y}}{{\left(x-b\right)}^{2}+c^{2}+e^{2}+B_{y}^{2}}+d$$

where *b* is the light shift, *a*, *c*, *d* and *e* are the fitting coefficients. After compensating the residual magnetic field in all three directions, a 15 nT bias magnetic field was utilized in the *y* direction and the magnetometer’s output signal was measured by applying *B*_z_ values from −1000 nT to 1000 nT. According to the fitting formula (16), the light shift *L*_z_ value was approximately 2 nT, when the pumping power was 50 mW. The test result of the light shift *L*_z_ is shown in Figure 11. The light shift was equivalent to a virtual magnetic field, which caused the residual magnetic field to increase while the sensitivity was reduced.

**Figure 12:**
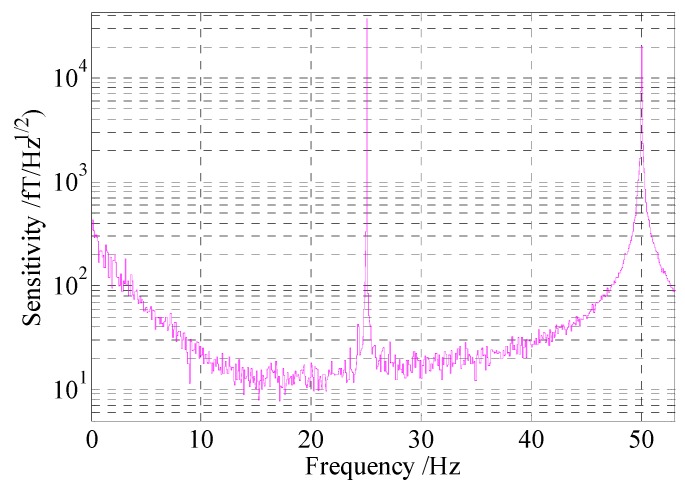
Optimal sensitivity of the atomic magnetometer based on suppressing the noises.

After suppressing the noises by the methods presented in this paper, the optimal sensitivity of 13 fT/Hz^1/2^, which was mainly limited by the Johnson noise, was achieved, as shown in Figure 12. The bandwidth of our SERF atomic magnetometer was approximately 40 Hz. In addition, there is a significant noise increase observed below 10 Hz. Unfortunately, at present, we don’t know the cause of this problem. In future, we will attempt to clarify this phenomenon through further study.

## 5. Conclusions

The principle of the SERF atomic spin magnetometer was introduced in this paper. According to the established output signal, the factors that influence he output signal and sensitivity were analyzed. The magnetic field, including the residual magnetic field and the light shift, the temperature and the light field were the main factors influencing the sensitivity. The residual magnetic field could be compensated and the light shift can be suppressed by adjusting the laser frequency to the light shift zero point. The operational temperature, which is related to the atomic density number, the polarization rate projection and the relaxation rate, should be 180 °C. The stabilization systems of the frequency and the power were presented, and the power stabilization experimental results showed that the power fluctuations could be controlled in 10%. Finally, contrast experiments on the sensitivity were implemented, and the results indicated that after suppressing the noises, an optimal sensitivity of 13 fT/Hz^1/2^ was successfully achieved.
